# Computational Fluid Dynamics Reveal Hydromechanical Adaptations of *Ceriodaphnia dubia* as an Inducible Defence Against the Carnivorous Plant *Utricularia* × *neglecta*

**DOI:** 10.1002/ece3.74244

**Published:** 2026-08-28

**Authors:** Eva M. Schulenberg, Patrick Piske, Marija Tomcic, Linda C. Weiss, Martin Horstmann

**Affiliations:** ^1^ Department of Animal Ecology, Evolution and Biodiversity Ruhr University Bochum Bochum Germany; ^2^ Global Change Ecology Ruhr University Bochum Bochum Germany

**Keywords:** carnivorous plants, computational fluid dynamics, *Daphnia*, hydromechanics, inducible morphological defence, suction feeding

## Abstract

Water fleas of the family Daphniidae are strong ecological interactors in many lentic ecosystems. As the most abundant filter feeders in these systems, they form a crucial link between primary production and higher trophic levels. Due to this central role in freshwater food webs and their capacity to react to a range of environmental changes with phenotypic plasticity, Daphniidae are widely regarded as model organisms in ecological research. They form inducible defences, representing a form of phenotypic plasticity, which reduces an organism's vulnerability to many animal predators. Just recently, the first inducible defence against a carnivorous water plant, the Southern Bladderwort, *Utricularia* × *neglecta*, was observed in *Ceriodaphnia dubia* , coinciding with a significant reduction in predation rate. This species' defensive traits are not yet fully understood. *C. dubia* was observed to swim more slowly, reducing contacts. Furthermore, its body is more slender. This is counterintuitive, as the plant is gape‐limited, rendering outgrowing that gape intuitive. Using computational fluid dynamics (CFD), we followed a new hypothesis on the inducible defence's function. We simulated the drag forces acting on daphniids in the typical and the *U*. × *neglecta*‐exposed morph. Our results indicate a decreased drag on the plant‐exposed morph, likely acting in concert with a reduced probability of contact with the plant's suction‐eliciting trigger hairs due to proportionally smaller second antennae. We therefore propose a protective inducible defence mechanism based on altered morphology in combination with modified hydromechanical properties. This newly discovered defensive strategy of hydromechanical adjustments adds another example to *Daphnia*'s reputation as an ecoresponsive organism.

## Graphical abstract

This study investigates an inducible defence of *Ceriodaphnia dubia* against the carnivorous plant *Utricularia* × *neglecta* using computational fluid dynamics. Simulations comparing the typical and plant‐exposed morph show that the induced morphology reduces drag and, together with proportionally smaller second antennae, likely lowers the probability of triggering the plant's suction traps. The results suggest a novel defensive strategy based on hydromechanical morphological adjustments.

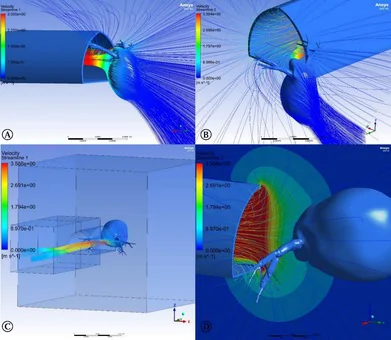

## 1 Introduction

In many ecosystems, predation plays a major role (Jeschke et al. 2022). Therefore, some prey species have evolved inducible defences, forming non‐permanently expressed alterations of morphology, behaviour or life‐history, thwarting predation (Tollrian and Harvell 1999). Inducible defences are especially known in freshwater crustaceans of the family Daphniidae (Lass and Spaak 2003; Weiss and Tollrian 2018). These members of the freshwater zooplankton are important links between primary production and higher trophic levels, as they filter‐feed on phytoplankton and fall prey to insect larvae, other crustaceans or fish (Lampert 2006). Within the Daphniidae, the formation of morphological alterations, such as helmets, elongated tail spines and thorns, as well as hidden morphological defences in the form of increased stability, have been reported (Laforsch et al. 2004; Rabus et al. 2013; Kruppert et al. 2017; Diel et al. 2020, 2021). Moreover, behavioural alterations like diel vertical migration, changes in swimming velocity or life history alterations such as shifts in the number of offspring, offspring size and age at maturity are typical inducible defences in this family (Lampert 1987; Lüning 1992; Stibor 1992; Stibor and Lüning 1994; Langer et al. 2019, 2021).

**FIGURE 1:**
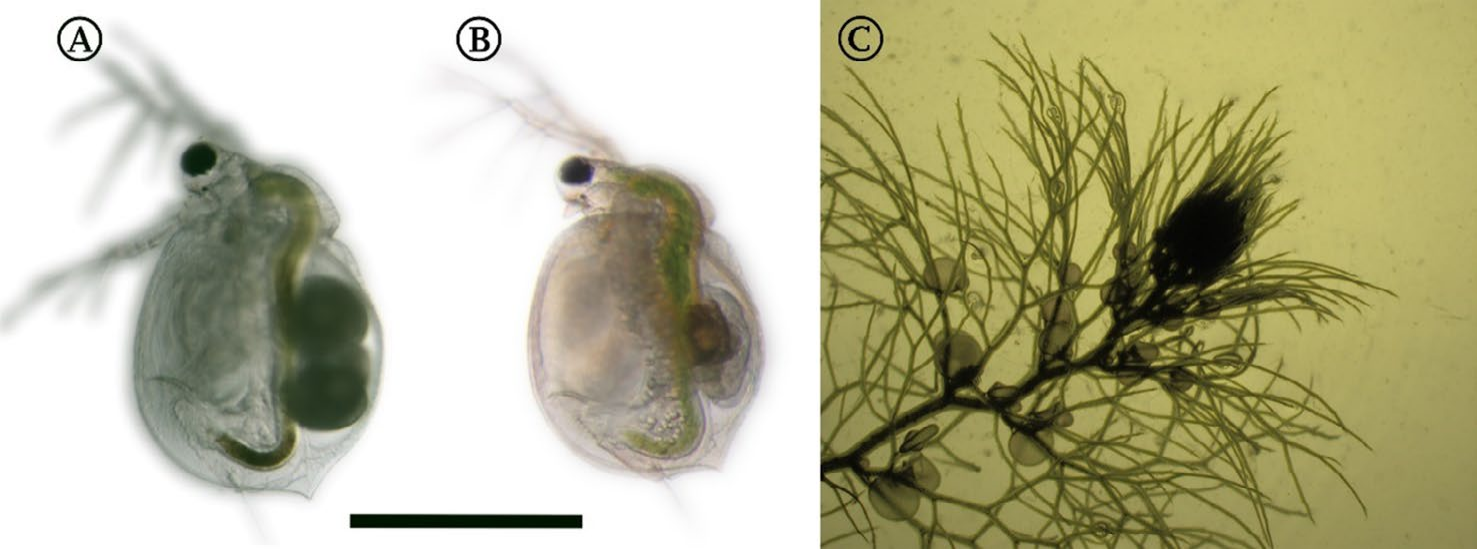
*Ceriodaphnia dubia* and *Utricularia* × *neglecta*. (A) Adult control *C. dubia* . (B) Adult defended *C. dubia* of the same age. Scale bar 1 mm. (C) *Utricularia* × *neglecta*, shoot tip and leaves with bladders.

Only recently, inducible defences of *Ceriodaphnia dubia* (Figure 1A,B) were described in response to carnivorous plants, namely the Southern Bladderwort, *Utricularia* × *neglecta* (Kruppert et al. 2022; Figure 1C). These plants use suction traps located on their leaves to prey on daphniids as well as other prey organisms (Darwin 1875; Vincent, Weißkopf, et al. 2011; Poppinga et al. 2016, 2017). The traps' fast suction movement, called the ‘firing’ of the traps, which either occurs spontaneously or is elicited by trigger hairs on the trap door, results in a water flow at velocities of approximately 4 m/s for about 5–9 ms close to the trap entrance (Harms 2002; Adamec 2011; Vincent, Roditchev, and Marmottant 2011; Poppinga et al. 2016, 2017). Prey that is sucked in experiences accelerations of up to 2800 g (Poppinga et al. 2017).

Accordingly, the trap door opening is one of the fastest plant movements known so far (Poppinga et al. 2013, 2016). To achieve such high velocities, which are hypothesised to help overcome the viscosity of water at low Reynold's numbers (Singh et al. 2020), glands on the inside of the traps actively pump out water (Vincent, Weißkopf, et al. 2011). This leads to a deflation of the traps, which creates elastic loading in the trap's walls (Joyeux et al. 2011). The trap door remains closed during this whole process. When the trap is fired, the stored elastic energy in its walls is released, causing the trap to inflate rapidly with water through a strong inbound current (Joyeux et al. 2011; Westermeier et al. 2017).

Water velocities around the trap entrance are exponentially reduced with distance to the narrowest point, as is typically observed around the mouth of other typical suction feeders, like fish (Day et al. 2005; Van Wassenbergh and Aerts 2009; Singh et al. 2020). Therefore, the drag forces acting on the prey are a relevant factor influencing whether or not it is captured. Prey organisms with more drag are accordingly more prone to being captured.

Typically, morphological defences against gape‐limited predators are an increase of body dimensions to simply outgrow the predator's gape. This anti‐lock‐and‐key mechanism (Dodson 1974) was, for example, observed in *D. lumholtzi* , which elongates its body appendages in the presence of fish, or in *D. magna* , which widens its body dimensions in the presence of *Triops* (Engel and Tollrian 2009; Rabus and Laforsch 2011; Engel et al. 2014). As *Utricularia* is also a gape‐limited predator, prey too large to fit through the trap entrance cannot be captured. However, previous literature found that *C. dubia* grows even slower in the predators' presence. The only growth in *Utricularia*‐exposed animals appears at the location of the fornices, a protrusion between the head shield and the carapace (Benzie 2005). The fornices are 37% wider than in non‐exposed animals (Kruppert et al. 2022). Potentially, investing in increased body size may be ineffective, since *C. dubia* is too small to successfully outgrow the predators' gape, which is already hypothesised in another predator–prey system (Santangelo et al. 2011). For *C. dubia* and *Utricularia*, it was furthermore speculated that the elongated fornices hinder the inflow of the prey into the trap lumen, whereas the smaller body reduces suction forces. Moreover, in case of a larger body, the trap entrance could be fully blocked. Consequently, the full suction forces would act on the prey body with potentially lethal injuries. Due to the measured average trap entrance size of 495 μm (±166 μm SD) height and 613 μm (±147 μm SD) width, defended animals with a lateral width of on average 475 μm, are definitely in the prey range (Kruppert et al. 2022). Because *U*. × *neglecta* traps vary in gape size, the total number of traps that are able to catch *C. dubia* even increases with decreasing animal size, since they will also fit into smaller‐sized traps (Friday 1991).

Therefore, not only the smaller body size as such may be relevant for the reduced predation rate, but also the drag forces acting on the prey. Here, also the so‐called antenna parachute, which is responsible for the power strokes as well as the deceleration of the sinking in daphniids, is relevant (Hantschmann 1961; Jacobs 1967; Skipper et al. 2019).

To test the influence of morphological changes on the drag forces acting during suction events, we reconstructed average three‐dimensional models of the control and the defended morph, and simulated flow effects using Computational Fluid Dynamics (CFD). With the help of this method, we checked whether smaller forces act on the defended daphniid body and in which position of the animals relative to the trap entrance, suction forces were highest. Poppinga et al. (2017) found that most *C. dubia* that were sucked into the trap were located laterally in front of the trap entrance. Some had their head oriented towards the trap, and only one individual had the carapace touching the trigger hairs, thus eliciting a firing of the trap. Using a method like CFD, we can objectively measure the forces acting on the body, without relying on the animals' behaviour in the experiment. As drag forces decrease with distance to the suction trap origin (Holzman et al. 2012), we hypothesise the drag forces to be highest in positions directly in front of the trap entrance. We furthermore hypothesise that among these positions close to the trap entrance, animals positioned upright experience the highest drag, as in this position, the antenna parachute and body volume cover most of the trap entrance in close distance. We further hypothesise the drag to be substantially lower for the defended *C. dubia* , rendering their survival chances higher. We also hypothesise the antenna parachute to be smaller in defended individuals, reducing drag not only with smaller bodies but also with smaller ‘paddles’ that function as sails in the case of suction feeding.

## 2 Methods

### 2.1 Replication Statement

**Table:** 

Scale of inference	Scale at which the factor of interest is applied	Number of replicates at the appropriate scale
Individuals	Morph	Morphology: 6 replicates, from which the following numbers could be used: control Day 3: 20 individuals from 5 jars; defended Day 3: 19 individuals from 3 jars; control Day 6: 24 individuals from 6 jars; defended Day 6: 13 individuals from 3 jars.
Individuals	Model	Simulations: 6 positions for control (the same) 6 positions for defended.
Morph	Individuals	For the 3D models: 13 for control, 8 for defended.

### 2.2 Culture and Induction

We worked with *Ceriodaphnia dubia* , clone S04, which originated from a shallow pond in Gelsenkirchen, Germany (51°30′17.9″ N, 7°04′58.7″ E), and has been cultured in our laboratory for several years. Culture and experiments were carried out in 1 L glass jars (Weck GmbH und Co. KG), filled with the medium ADaM (Klüttgen et al. 1994) at 20°C ± 1°C and a 16 h:8 h light to dark cycle. *C. dubia* was fed *ad libitum* with a suspension of *Tetradesmus obliquus* and *Nannochloropsis* spec. every other day.

Furthermore, we used *U*. × *neglecta* initially purchased at Gartenbau Thomas Carow (Nüdlingen, Germany) and cultivated in the Department of Animal Ecology, Evolution and Biodiversity of the Ruhr‐University Bochum, Germany. Plants were kept in 25 L glass aquaria filled with deionised water and dried *Carex* leaves as substrate. Aquaria were placed under an LED light source (Dennerle Nano Power 5.0). The *U. × neglecta* culture was kept above room temperature (at 25°C ± 1°C and a 16 h:8 h light to dark cycle), keeping the plants continuously growing and producing new traps.

For the induction of inducible morphological defences, we selected females carrying eggs in the last embryonic stage. We divided them into two groups and placed them in net cages (height 12 cm, diameter 7 cm) with a mesh width of 125 μm (Hydrobios, Kiel, Germany) within 1 L glass jars filled with ADaM, as in culture. In each induction treatment, one approximately 15 cm plant shoot was placed outside the net cage and fed with 25 juvenile *C. dubia* to enhance defence expression (Kruppert et al. 2022). This setup was replicated 6 times. We determined the body length, width and antenna length of the offspring on the third and sixth day. On the sixth day, the offspring reached maturity, that is, inserted eggs into the brood pouch. For the three‐dimensional modelling and simulation of suction, only models of these adult *C. dubia* were used, carrying their first eggs in the brood pouch.

### 2.3 Measurements of Morphological Traits

**FIGURE 2:**
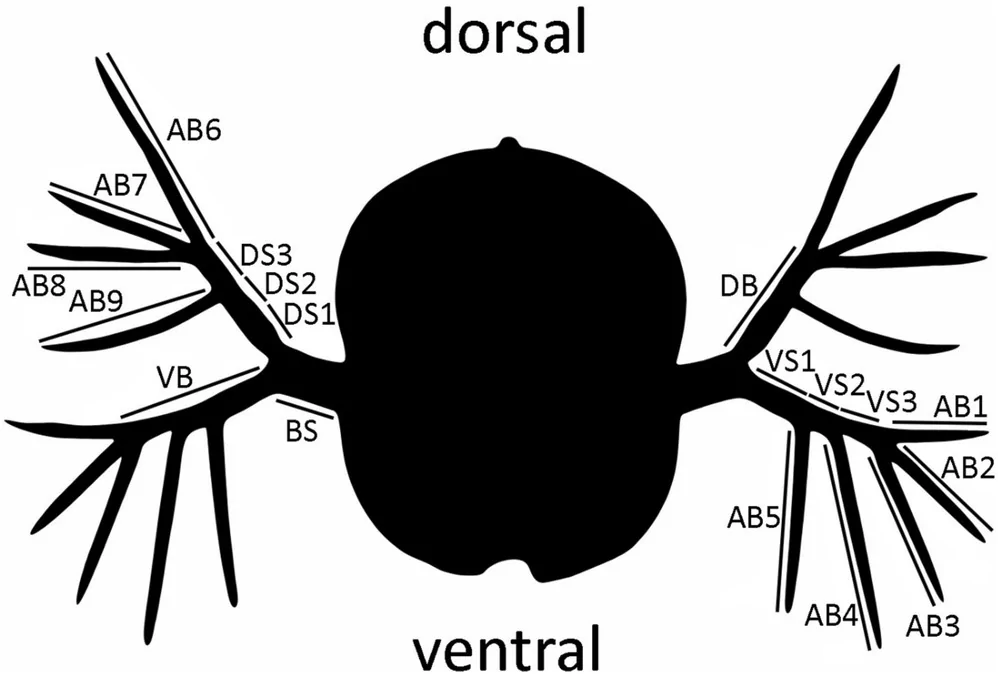
Schematic drawing of the antenna parachute in *Ceriodaphnia dubia* . Marked are the basal segment (BS), the dorsal (DB) and ventral branch (VB), consisting of three segments each, as well as the nine antenna bristles (AB1‐9). The total length of the ventral parachute is measured as the length of the ventral segments (VS) 1–3 plus the terminal antenna bristle (AB) 1. The total length of the dorsal parachute was measured likewise (dorsal segments (DS) 1–3 plus terminal antenna bristle (AB) 6).

In order to measure body length and body width, animals were transferred onto an object slide and photographed using a stereomicroscope (SZX 16, Olympus), equipped with a digital camera (Vidmess, Thalheim Spezialoptik, Pulsnitz, Germany). For measurements of the antennae, animals needed to be photographed in their resting position during swimming. This meant pictures had to be taken from above. To reduce swimming velocities and facilitate taking sharp pictures without motion blur, we reduced the animals' temperature by transferring them in pipette tips glued onto object slides. These were stored on ice for at least 10 min. For the actual measurements, these object slides were transferred into a petri dish filled with ice‐cold water and ice. Photos were taken when the animals were in the focus layer and in the sinking phase of their locomotion cycle (Skipper et al. 2019) spreading their antennae at approximately a 90° angle to the body. We did not differentiate between the left and right antenna. In total, we measured traits of 20 individuals from 5 jars for the combination control/Day 3, 19 individuals from 3 jars for the combination defended/Day 3, 24 individuals from 6 jars of the combination control/Day 6, and 13 individuals from 3 jars of the combination defended/Day 6. In detail, the lengths of the basal segment (BS), the three segments of the ventral (VS1‐3), as well as of the dorsal branch (DS1‐3) and the five antenna bristles (AB1‐5) at the ventral and the four antenna bristles at the dorsal branch (AB 6‐9) were measured (Figure 2). For the statistical analysis shown in this manuscript, we present the span of the dorsal and ventral parachute, each consisting of the respective branch segments and the terminal bristle, the length of the basal segment and the sum of the lengths of antenna bristles' with respect to treatment. We also calculated relative lengths by dividing each value by the body length of the respective animal. We analysed all morphological data using generalised mixed models glmmTMB (Brooks et al. 2017) in R (version 4.3.1, R Core Team 2021) and plotted using the packages ggplot2 (Wickham 2016) and ggpubr (Kassambara 2022). Each model for the trait in question was modelled as trait ~ treatment × day + (1|replicate), using the jar/replicate as random factor. Pairwise comparisons were calculated after modelling using the package emmeans (Lenth 2022).

### 2.4 3D‐Modelling

For the analysis of three‐dimensional models using CFD, we used 3D data already published in Kruppert et al. (2022). We scanned one half of each morph, then—assuming bilateral symmetry—mirrored this half to obtain a full model of the animals and closed any holes between the halves, as also described in Horstmann et al. (2022). For the creation of a 3D antenna model, we sacrificed several adults using 4% formaldehyde in phosphate buffered saline (PBS‐) buffer solution, in which we placed the animals for a few seconds. To avoid overfixation, we immediately washed the animals with PBS five times for 2 min and stored them in PBS at 4°C overnight. Using fine tweezers and a needle, the antennas were carefully separated from the body under a stereo microscope the next day. They were transferred to a 1.5% Congo red solution and dyed overnight, before rinsing them five times in PBS again. Scanning was conducted with a confocal laser scanning microscope (Leica TCS SP5II) using a slice thickness of 1 μm and a resolution of 1024 × 1024 pixels. As described in Horstmann et al. (2018), we extracted the surface of a single well‐preserved antenna per treatment using MorphoGraphX (de Reuille et al. 2015). This antenna was then virtually attached to the respective three‐dimensional model of the defended or control body using Blender (version 3.0.1, Blender Foundation, Blender Institute Amsterdam) in the typical resting position, spread at an angle of about 90° to the body.

### 2.5 Simulation of Suction Forces

**FIGURE 3:**
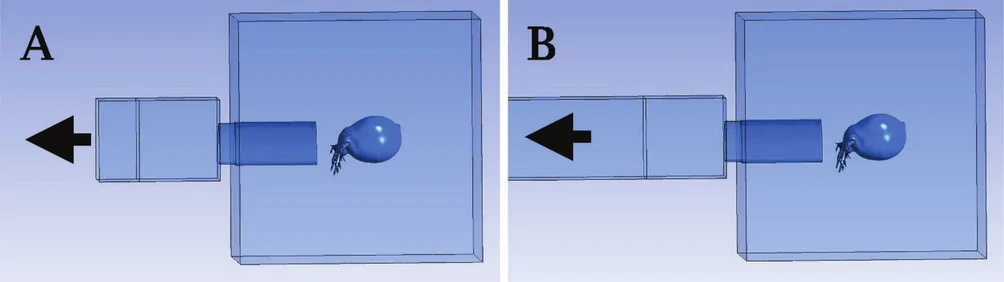
Simulation setup for calculating the suction of an *Utricularia* × *neglecta* trap. (A) Model positions before simulation. The arrow indicates the movement of the most left wall of the smaller cube. (B) Model during peak suction forces. The arrow indicates that the most left wall was accelerated to the left to induce a negative pressure difference in the smaller cube, eliciting an inflow of water from the surroundings into the trap entrance for pressure equilibration.

For the simulation of suction forces, we modelled a fluid space of 2 mm by 2 mm using Ansys ICEM CFD (ANSYS 2020 R1, ANSYS Inc., Canonsburg, PA, United States), in which we positioned the model of the control and defended *C. dubia* at five locations and with different orientations. Using the average 3D‐models based on the shape of 13 control and 8 defended individuals, natural morphological variation was considered without the time‐consuming step of individual simulations. On one side of the fluid space, we integrated a tube with the dimensions of an average *Utricularia* × *neglecta* trap entrance (width 613 μm, 495 μm height, Kruppert et al. 2022) and the typical flat trap threshold and bow‐shaped entrance. This tube was modelled to end in a smaller cube of 1 mm edge length. In this cube the rear wall was modelled as moveable by a mathematical function in Ansys CFX Pre (Figure 3). This function was adjusted in a way similar to the parameters of the suction event of *U*. × *neglecta*, that is, a suction duration of about 8 ms and a maximal fluid velocity of 4 m/s (Poppinga et al. 2017). The six walls of the large cube were defined as ‘opening’ with a static pressure of 0 Pa. The surface of the *Ceriodaphnia dubia* models was defined as ‘wall’ with the property ‘No Slip Wall’ and the option ‘Smooth Wall’. All other surfaces were simulated as ‘walls’ with the property ‘No Slip Wall’ and the option ‘Smooth Wall’ as well. Only the surfaces connecting the moving wall with the rest of the small cube were defined as ‘No Slip Wall’, ‘Wall Velocity relative to Boundary Frame’ and ‘Smooth Wall’, as it is going to be stretched during the simulation. The moving wall itself was defined as ‘No Slip Wall’ and the option ‘Specified Displacement’ and ‘Smooth Wall’. The formula for the movement was defined as follows:

ft=−1.8505ttime01+e,1+7×1mm,with'time'representing1sinaseparateexpression

The models were built of 6.5 and 1.3 million tetrahedrons for the control and defended model, respectively. As fluid we used water at 25°C. As further pre‐set options, we chose the simulation with the k‐Epsilon turbulence model. We used the Ansys solver and adjusted the convergence criterion to 1 × 10^−6^ and a maximum of 30 loops. The suction process was then simulated, and forces acting on the models in *x*, *y* and *z* directions were calculated in 31 time steps in Ansys CFX Solver, with time step 1 being the start situation. The resulting total forces were determined in Ansys CFD‐Post.

**FIGURE 4:**
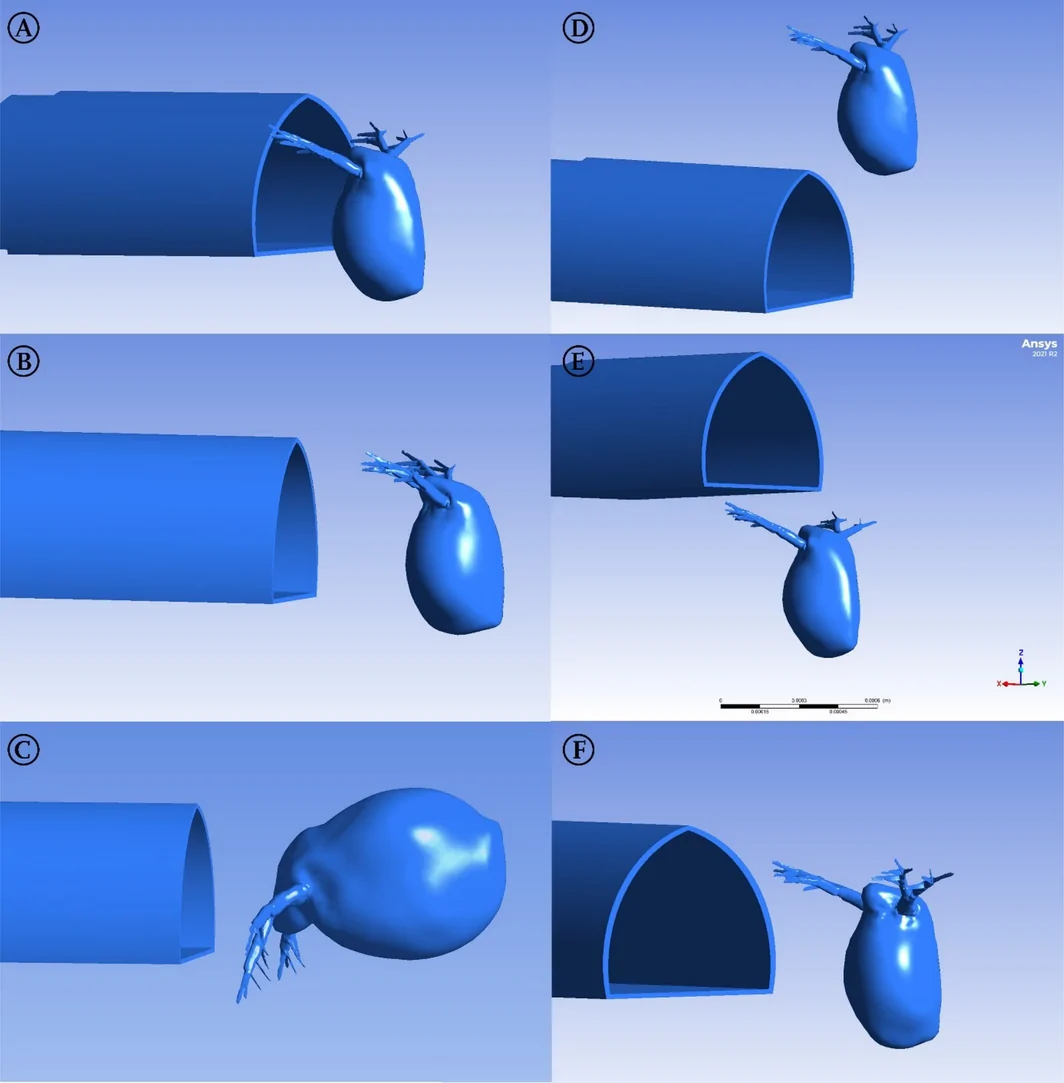
Positions of *C. dubia* models relative to the virtual trap entrance. Models of average defended and control *C. dubia* were positioned at different distances and positions relative to the virtual trap entrance. Suction velocity of the trap was adjusted to 4 m/s, which occurred as peak velocity within a suction duration of 8 ms. We modelled animals in front of the trap entrance (A), with some distance (B), forward‐swimming daphniids (C), as well as daphniids above (D), beneath (E) and lateral to the trap entrance (F). Shown are models of the defended morph, except for C, which depicts the control morph for size comparison to the trap entrance.

In detail, we positioned the models directly in front of the trap entrance (Figure 4A), with a distance of the ventral body parts of about 200 μm from the trap entrance. Furthermore, we positioned the models with a larger distance of 400 μm to the trap entrance (Figure 4B). At this location, we also rotated the model about 90° with the antennae facing the trap entrance, also with the main body (in that case the head) in a distance of 400 μm to the trap entrance (Figure 4C). We furthermore positioned the models above (Figure 4D) and below the trap entrance (Figure 4E) as well as next to it (Figure 4F). Our results are available online in a Zenodo repository (https://doi.org/10.5281/zenodo.18195939).

## 3 Results

### 3.1 *Ceriodaphnia dubia* Body Length, Body Width and Antenna Length

**FIGURE 5:**
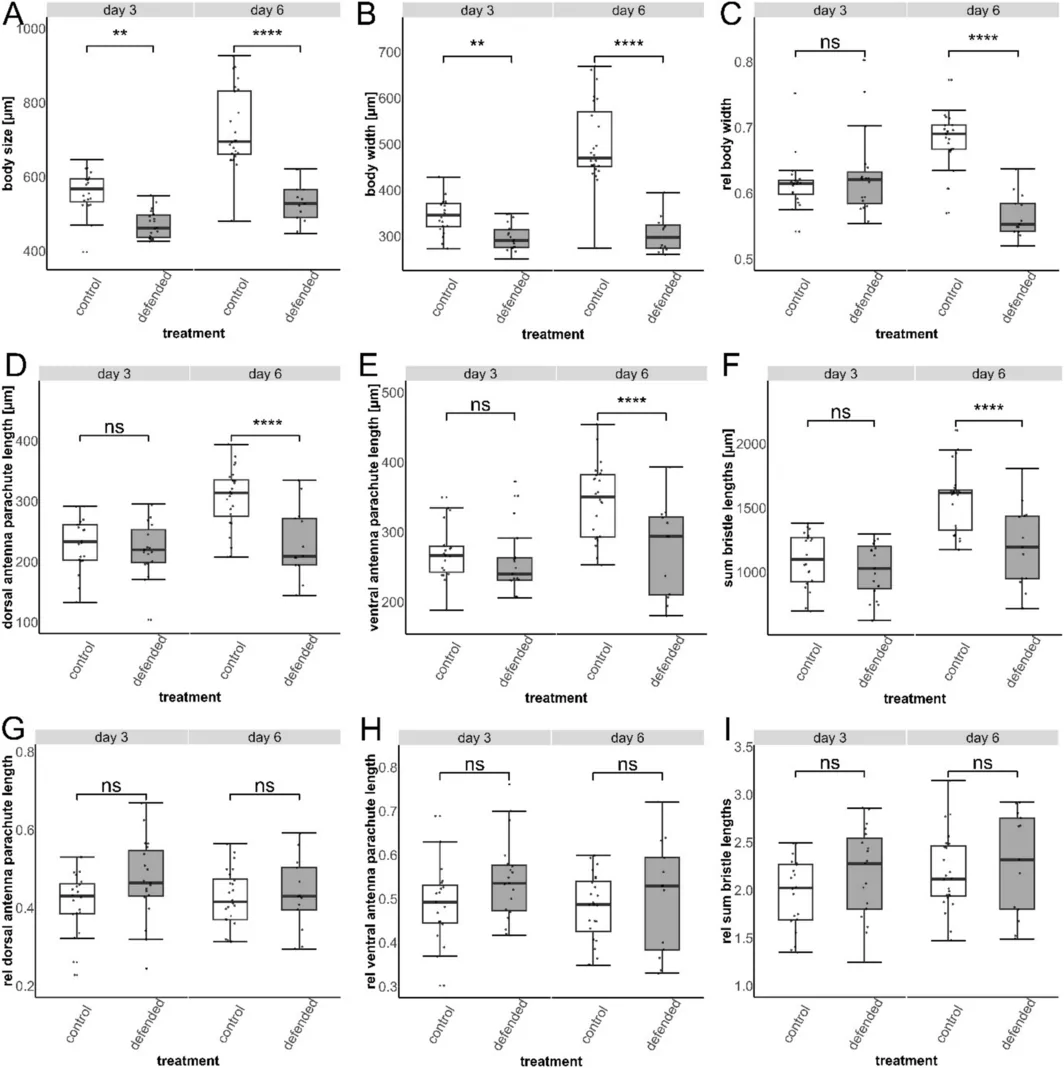
Morphological alterations in *Ceriodaphnia dubia* reared under control and *Utricularia* × *neglecta* conditions (defended), three and 6 days after hatching. (A) Body length. (B) Body width. (C) Relative body width, normalised to body length. (D) Span of the dorsal antenna parachute (dorsal segments + antenna bristle 6). (E) Span of the ventral antenna parachute (ventral segments + antenna bristle 1). (F) Summed length of all antenna bristles. (G) Relative span of the dorsal antenna branch (relative length of dorsal segments + relative length of antenna bristle 6). (H) Relative span of the ventral antenna branch (relative length of ventral segments + relative length of antenna bristle 1). (I) Summed length of all antenna bristles normalised to body length. Asterisks indicate significance levels according to the conducted pairwise comparisons carried out with the emmeans‐package after modelling the data.

Already on Day 3, body lengths of control (560 ± 56.6 μm) and defended *C. dubia* (471 ± 36 μm) differed significantly (*p* < 0.0001, Figure 5A). This difference between treatments increased to Day 6 (*p* < 0.0001). While control individuals (728 ± 105 μm) grew by about 170 μm during these 3 days, defended individuals (529 ± 53 μm) grew about 60 μm on average. Body width was significantly different between control (344 ± 38 μm) and defended *C. dubia* (295 ± 30 μm) on Day 3 (*p* < 0.001), and on Day 6 (control 500 ± 90 μm, defended 300 ± 40 μm, *p* < 0.0001, Figure 5B). While the defended individuals had no significant alteration in relative body width on Day 3 (control = 0.615 ± 0.04, defended = 0.626 ± 0.06, *p* = 0.62), it was reduced on Day 6 (control = 0.683 ± 0.04, defended = 0.566 ± 0.03, *p* < 0.0001, Figure 5C).

Based on the average models, we determined the average volume of both morphs as 0.000131 cm^3^ for the control model and 0.000026 cm^3^ of the defended model, which is a five times higher volume in the control morph.

Parallel to the reduced body size, antenna lengths were also reduced in defended individuals. While on Day 3 no significant differences were observed for the absolute lengths of the dorsal (control = 229 ± 42 μm, defended = 222 ± 45 μm, *p* = 0.56, Figure 5D) nor the ventral (control = 264 ± 48 μm, defended = 256 ± 47 μm, *p* = 0.19, Figure 5E) nor the basal branch (control = 92 ± 22 μm, defended = 87 ± 22 μm, *p* = 0.46), the antenna sizes differed significantly for the dorsal (control = 305 ± 50 μm, defended = 231 ± 56 μm, *p* < 0.001, Figure 5G), ventral (control = 345 ± 52 μm, defended = 266 ± 69 μm, *p* < 0.01, Figure 5H) and basal branch (control = 120 ± 30 μm, defended = 93 ± 18 μm, *p* < 0.01) on Day 6, with control specimens having longer antennas.

While absolute antenna lengths differed in *C. dubia* exposed to the control and defended treatment on Day 6, relative antenna lengths mostly did not differ (dorsal segments Day 3: control = 0.412 ± 0.08, defended = 0.472 ± 0.10, *p* = 0.04, Day 6: control = 0.424 ± 0.08, defended = 0.436 ± 0.09, *p* = 0.72; ventral segments Day 3: control = 0.477 ± 0.1, defended = 0.544 ± 0.09, *p* = 0.053, Day 6: control = 0.479 ± 0.07, defended = 0.503 ± 0.12, *p* = 0.56; basal segment Day 3: control = 0.168 ± 0.05, defended = 0.187 ± 0.05, *p*‐value = 0.32, Day 6: control = 0.166 ± 0.04, defended = 0.179 ± 0.04, *p* = 0.32).

The sum of absolute antenna bristle lengths was not significantly different between treatments on Day 3 (control = 1095 ± 210 μm, defended = 1018 ± 204 μm, *p* = 0.18), while we observed a significant difference on Day 6 (control = 1565 ± 243 μm, defended = 1203 ± 310 μm, *p* < 0.01, Figure 5F). The sum of relative antenna bristle lengths was not significant on any of the days compared (Day 3: control = 1.963 ± 0.36, defended = 2.172 ± 0.46, *p* = 0.12; Day 6: control = 2.18 ± 0.39, defended = 2.273 ± 0.53, *p* = 0.63, Figure 5I), which replicates the observations for the lengths of antenna segments.

### 3.2 Drag Forces at Different Positions

**FIGURE 6:**
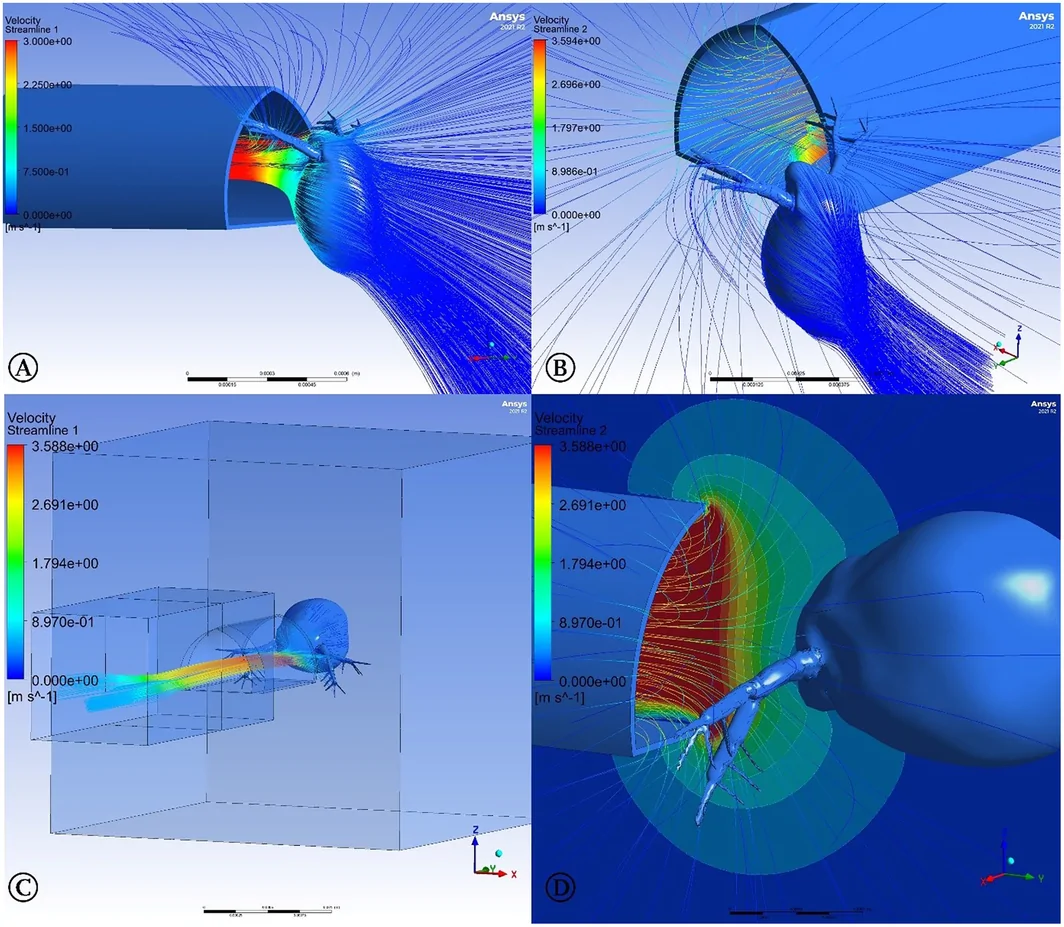
Streamlines simulated during the suction event at different positions relative to the trap entrance. All images show the control morph. (A) Model in front of the simulated trap entrance. In this position, the highest drag forces were measured. (B) Model lateral to the trap entrance. (C) Model facing the trap entrance with head and antenna. (D) Same model as in (C), but with an added plane cutting sagittally through the animal, showing water velocities around the trap entrance.

**FIGURE 7:**
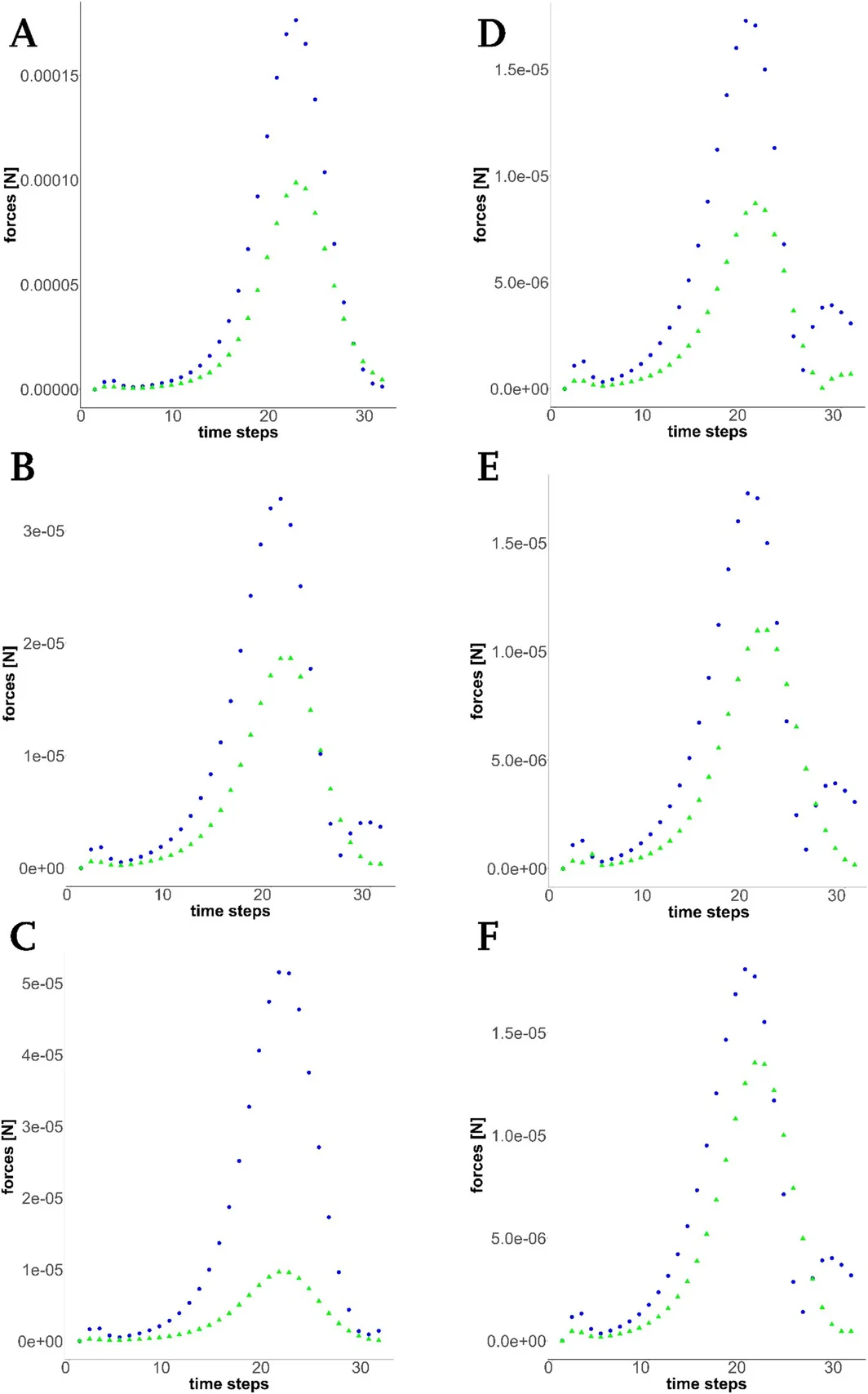
Total forces acting on the model of control (blue dots) and defended (green triangles) *C. dubia* positioned at the simulated trap entrance during the 30 steps of simulated suction. (A) Models positioned close to the virtual trap entrance. The defended model experienced half of the drag of the defended model at about time step 23 (approximately 6 ms), which is immediately before the deceleration of the water flow stops. (B) Models positioned with some distance to the simulated trap entrance. The defended model is affected by about half of the drag forces compared to the control. (C) Models positioned at the same position as in (B), but rotated by 90°, antennas towards the simulated trap entrance. This rotation increases drag forces acting on both models, with forces acting on the defended being about 20% of the control. (D) Models positioned above the simulated trap entrance. Defended model experiencing half of the forces. (E) Models positioned beneath the simulated trap entrance. The defended model experiences about a third of the drag forces of the control. (F) Models positioned laterally of the simulated trap entrance. The defended model experiences about 2/3 of the forces compared to the control.

The simulation of the suction of the trap after positioning the 3D models of control and defended *C. dubia* at different locations relative to the trap entrance yielded different drag forces acting on the models (Figures 6 and 7). In general, the highest drag forces occurred in the central suction phase when peak velocities of 4 m/s were produced at the simulated trap entrance. In defended *C. dubia* , highest drag forces were observed in the simulation with the 3D models positioned in front of the trap entrance and the lowest for models positioned laterally of the trap entrance. The same was observed in models of control *C. dubia* . At all time points in the main drag phase, the model of defended *C. dubia* experienced less, in many cases only half or less, of the drag forces acting on the control model.

#### 3.2.1 Models in Front of the Trap Entrance

The control as well as the defended animal's model experienced almost no drag forces in the first 10 time steps, which resembles the first 2.5–3 ms of beginning trap suction (Figure 7A). Beginning at time step 10, drag forces rise steeply, especially in the model of control *C. dubia* . A maximum is reached at time step 22, where the control model experiences forces of 1.76 × 10^−4^ N and the defended model of 9.88 × 10^−5^ N, which is about half the drag force of the control model. After that maximum for both models, forces again steeply decline.

#### 3.2.2 Models of Upright *C. dubia* With Some Distance

Both *C. dubia* models experience very low drag forces over about 12–15 time steps (Figure 7B). Until time step 21 (5.6 ms) in control and time step 22 (5.9 ms) in defended *C. dubia* , forces rise to the maxima of 3.28 × 10^−5^ N and 1.87 × 10^−5^ N, respectively. Again, drag forces in the defended model are about half the amount of the control model. Drag forces fall to close to zero within the next time steps, with the control model again experiencing a local maximum at time step 29 with 4.05 × 10^−5^ N.

#### 3.2.3 Models of Forward Swimming *C. dubia* With Some Distance

In this simulation, the largest differences between control and defended models occurred (Figure 7C). While both models have their maximum forces at about the same time step 22 (control, 21 in defended), the height of the maximum varies strongly. While the defended model experiences drag forces of 9.64 × 10^−6^ N, the force on the control model is 5.15 × 10^−5^ N. This represents a difference of approximately five times between the two models.

#### 3.2.4 Models of *C. dubia* Above the Trap Entrance

Both models show a small local maximum already at time steps 2 and 3 (Figure 7D). The overall maximum is reached at time step 20 for the control model at 1.73 × 10^−5^ N and at time step 21 for the defended model at 8.71 × 10^−6^ N. Drag forces acting on the defended model are halved compared to the defended model. After a steep decline, both models show increasing drag forces after time step 26. Here, the local maximum of 3.92 × 10^−6^ N in control models is higher than the maximum in defended models with 6.79 × 10^−7^ N.

#### 3.2.5 Models of *C. dubia* Beneath the Trap Entrance

Similarly to the models simulated above the trap entrance, also in the models positioned beneath, a small local maximum is achieved in both models already at time steps 2 and 3 (Figure 7E). The overall maximum for the control *C. dubia* model with 1.73 × 10^−5^ N lies at time step 20. For the defended model, it is at time step 22 with 1.1 × 10^−5^ N. Therefore, the defended models experience about 60% of the drag forces acting on the control model. After the maximum, drag forces drop close to zero in both models, while the control model experiences a second local maximum with 3.92 × 10^−6^N in time step 29.

#### 3.2.6 Models of *C. dubia* Lateral of the Trap Entrance

Both models experience a local maximum at time steps 2–3 at 1.33 × 10^−6^ N in the control and 4.69 × 10^−7^ N in the defended model (Figure 7F). Drag forces rise to a maximum of 1.81 × 10^−5^ N in time step 20 in the control model and 1.35 × 10^−5^ N in time step 21 in the defended model. Accordingly, forces acting on the defended model are 75% of the ones acting on the control model. The forces calculated for this position are comparable to those of the position above the trap entrance. While the forces in the defended model asymptotically approach zero, the control model experiences a second local maximum with forces of 4.02 × 10^−6^ N at time step 29.

## 4 Discussion

Previous literature (Kruppert et al. 2022) showed *Ceriodaphnia dubia* reacting to the presence of *Utricularia* × *neglecta* with inducible alterations of behaviour, life history and morphology. However, especially the altered morphology observed in this and a previous study (Kruppert et al. 2022) is counterintuitive: While typically inducible morphological defences against gape‐limited predators comprise outgrowing the predator's mouth width, defended *C. dubia* showed reduced body dimensions (Caramujo and Boavida 2000; Rabus and Laforsch 2011; Kruppert et al. 2022). We were able to replicate these results, finding that the body length in defended animals was reduced to on average 84% of the control animals on Day 3 after hatching and to on average 73% on Day 6, indicating reduced growth. We also found similar or even stronger reductions in body width, which was reduced to 86% of the respective control animals on Day 3 and even 60% on Day 6. In addition, absolute antenna segment lengths were significantly reduced in defended animals on Day 6. When comparing the morphological alterations relative to body size, mostly no significant alterations could be found, indicating a more or less proportional decrease in the lengths of the antenna segments. Only the relative body width was significantly smaller on Day 6 after hatching, that is, in adult animals, hinting at a change in body shape in the presence of *U*. × *neglecta*.

While suction feeding in vertebrates has often been modelled using CFD, rather few approaches have been conducted to investigate hydromechanics in invertebrates or even plant suction (Day et al. 2005; Van Wassenbergh and Aerts 2009; Holzman et al. 2012; Singh et al. 2020; Koehnsen et al. 2021). With our simulations, we found that reduced body dimensions and a smaller antenna parachute clearly reduce drag forces. Hydromechanically, the antenna parachute can likely be treated as a continuous surface (Jacobs 1967). This is due to the low Reynold's number of 10–100 that are associated with daphniids (Wickramarathna et al. 2014). The antennae with their bristles and attached finer setae (not simulated) are likely to function like the wings of Thysanoptera, which aerodynamically form a closed surface, despite being discontinuous and built of microscopic hairy appendages (Lee and Kim 2021). While typically the wings and antennae are used to allow flight, respectively propulsion, strong currents can catch both structures, turning them into a ‘sail’ or parachute. Accordingly, the reduction of the antenna parachute, even if it is a decrease proportional to body size, reduces the surface area that can be caught by the suction current of an elicited *U*. × *neglecta* trap.

Depending on the position, drag forces were reduced morphologically by up to 81% in the phase of strongest suction for defended models positioned close to the trap entrance. In the position with the antennae facing the trap entrance, the defended model experienced only 20% of the drag forces compared to the control model. Under the assumption that higher drag increases the risk for *C. dubia* to be sucked into *U*. × *neglecta's* traps, the observed morphological alterations can be clearly beneficial in predator contact. This, of course, only holds true under the assumption that prey does not simply move with the surrounding water independent of its shape and density. While Wainwright and Day (2007) assumed this in their models, Van Wassenbergh and Aerts (2009) found that body morphology can indeed have an influence on the suction feeding of predators, though the effect is minimal.

Whether this small simulated alteration via altered morphology can be reassured in actual predation experiments is questionable (Van Wassenbergh and Aerts 2009). It can hardly be tested, as other effects cannot be separated from morphology. For example, the trap loading situation and therefore the pressure difference between trap lumen and environment, which can be up to 0.176 bar (Singh et al. 2011; Adamec and Poppinga 2016), is dependent on the time since the last suction event. Nevertheless, previous experiments found that all traps elicited by a prey *C. dubia* successfully captured its prey (Poppinga et al. 2017). This suggests that ‘danger zones’ (Poppinga et al. 2019) can be identified around the traps of *U*. × *neglecta*, which might be smaller for defended animals due to their lower drag forces. Earlier studies already found a significantly reduced predation of *U*. × *neglecta* on defended *C. dubia* (Kruppert et al. 2022). However, it remains unclear whether the reduced predation rate was caused by the avoidance behaviour in the presence of these plants, by the reduced chances of encounter with the trap due to slower swimming motions, or by the altered morphology (Kruppert et al. 2022).

With this study, we provide the first evidence that indeed the morphological alterations (overall leading to reduced body dimensions) have a notable impact on the drag forces exerted by suction‐feeding predators. The importance of the antenna parachute for suction events elicited by *U*. × *neglecta* was already indicated by the rotation of the prey organism into a head‐to‐trap orientation during the suction events (Poppinga et al. 2017). Although we did not model the movement of the trap towards the prey, that is, the ‘forward strike’ of the trap observed by Poppinga et al. (2017), or the movement of the prey towards the trap, forces were, independent of the position, always higher in control animals. As prey organisms experience velocities of up to 4 m/s and accelerations of up to 2800 g in a few milliseconds (Poppinga et al. 2017), comparatively high forces could be expected. Unfortunately, with our simulations, we could only model fixed animal positions, that is, the prey models were not sucked into the trap during the simulations, but remained stationary. This means simulated forces are just an approximation to the forces that would be experienced by real animals. For example, once in motion, velocity differences between the flowing medium and the model would be smaller than calculated in our simulation.

Previous literature assumed prey would behave as an element of the sucked fluid, in which case friction and therefore drag would be negligible (Muller et al. 1982). Moreover, modelling prey particles as infinitesimally small and as an element of the water is also only valid for neutrally buoyant prey, which *C. dubia* is not (i.e., it falls to the ground of the body of water quite fast if not actively moving). Models for small fish and their prey also showed that, in general, it is not justified to treat prey similar to particles of water, as shape can disturb the flow field and, accordingly, affect drag forces. Moreover, as Van Wassenbergh and Aerts (2009) described, shape can influence drag. As a consequence of the antennas, the form of the prey is highly heterogeneous in the case of *C. dubia* models. Consequently, different parts of the prey experience different amounts of drag. Previous studies often modelled with spheres or spheroids for simplification (Wainwright and Day 2007; Van Wassenbergh and Aerts 2009), potentially also explaining the quite strong effects in our investigation compared to these simulations. Furthermore, the size of prey relative to the mouth opening is relevant. If the prey is large compared to the opening that is the origin of suction forces, which is the case in our analysis, flow fields are affected more strongly (Van Wassenbergh and Aerts 2009). Nevertheless, even if differences between the investigated morphs are small, these alterations may affect the drag forces and therefore the ecological result of increased survival for defended *C. dubia* . Depending on the benefits of the defence, even small alterations can cause evolutionary advantages that is, a fitness increase of one morph over the other, which justifies the expression of such altered morphologies.

The strong response of *C. dubia* to the presence of *U*. × *neglecta* with morphological, behavioural and life history alterations indicates the presence of a substantial predation pressure by the plant. The observed inducible defences come at the trade‐off of reduced fertility (Kruppert et al. 2022). If the alterations were too costly or too ineffective, they would not be formed (Tollrian and Harvell 1999). Despite *U*. × *neglecta* trap contents typically containing many substrate‐bound prey organisms, which are believed to be guided towards the trap entrance via the appendages (sometimes called antennae) in front of the trap entrance, *C. dubia*, as a freely swimming daphnid, is regularly found as well (Meyers and Strickler 1979; Harms and Johansson 2000; Kruppert et al. 2022). Poppinga et al. (2017) showed that this species can elicit a firing of the trap during locomotion movements; however, prey may be so close that inducible defences are not protective. Still, spontaneous firings of the traps can also endanger the prey organisms. These suction events, which are not triggered by prey, but rather the pressure difference between the trap lumen and the environment, may, by chance, also affect prey (Adamec 2011; Vincent, Roditchev, and Marmottant 2011; Poppinga et al. 2016). In that case, prey is more likely to swim at some distance to the trap entrance and will therefore experience drag forces like we simulated them in the above, beneath and lateral positions. As mentioned, also in these positions we found differences between the control and defended animals. Especially as drag forces are highest close to the trap entrance (Holzman et al. 2012), the general reduction of drag forces through morphological alteration reduces the danger of being sucked into the trap lumen, independent of the position relative to the trap.

The observed alterations in drag forces due to smaller body dimensions may also have implications for other prey organisms of suction feeding predators. For example, *D. lumholtzi* defends against fish predation with a pointed helmet and elongated tail spine, increasing body span (Sorensen and Sterner 1992; Engel and Tollrian 2009; Engel et al. 2014). At the same time, it also shows a smaller body size (Horstmann et al. 2021). Potentially, this body shape also has the effect of reducing the drag forces, while, if successful suction occurs, the elongated appendages block the predator's mouth. The results for one clone of *Ceriodaphnia dubia* presented here are already convincing that one component of the observed successful morphological alteration may be drag reduction. Further investigations of other clones and other daphniid species are necessary to ascertain whether a general pattern of reduced body sizes is an effective response towards suction‐feeding predators. This promises to be a worthwhile objective for future studies, especially in the context of operational costs of inducible defences in daphniids.

## Author Contributions

**Eva M. Schulenberg:** methodology (equal), software (equal), supervision (lead), writing – original draft (equal), writing – review and editing (equal). **Patrick Piske:** data curation (equal), formal analysis (equal), investigation (equal), visualization (equal), writing – original draft (equal). **Marija Tomcic:** data curation (equal), investigation (equal), software (lead), visualization (equal). **Linda C. Weiss:** project administration (lead), resources (lead), supervision (supporting), writing – review and editing (supporting). **Martin Horstmann:** conceptualization (lead), funding acquisition (lead), methodology (equal), supervision (supporting).

## Funding

This work was supported by the Deutsche Forschungsgemeinschaft (DFG) under the grant number HO 7615/1‐1 (project number: 545843880).

## Conflicts of Interest

The authors declare no conflicts of interest.

## Data Availability

Morphological measurements, 3D‐models of the defended and control morph as well as tables giving the forces determined via Ansys simulations are deposited in a Zenodo repository: https://doi.org/10.5281/zenodo.18195939.
